# What patients really think about asthma guidelines: barriers to guideline implementation from the patients’ perspective

**DOI:** 10.1186/s12890-016-0346-6

**Published:** 2017-01-11

**Authors:** H. Lingner, B. Burger, P. Kardos, C. P. Criée, H. Worth, E. Hummers-Pradier

**Affiliations:** 1 Centre for Public Health and Healthcare, Biomedical Research in Endstage and Obstructive Lung Disease Hannover (BREATH), Member of the German Center for Lung Research (DZL), Hannover Medical School, Carl-Neuberg Strasse 1, 30625 Hannover, Germany; 2 Klinik für Psychosomatische Medizin, DIAKOVERE gGmbH - Henriettenstiftung, Hannover, Germany; 3 Group Practice & Centre for Allergy, Respiratory and Sleep Medicine, Frankfurt, Germany; 4 Department of Pneumology, Respiratory Care, Sleep Medicine, Evangelisches Krankenhaus Göttingen-Weende gGmbH, Bovenden-Lenglern, Germany; 5 Departments of Pneumology and Cardiology, Hospital Fürth, University Erlangen-Nürnberg, Fürth, Germany; 6 Department of General Practice, University Medical Center Göttingen, Göttingen, Germany

## Abstract

**Background:**

Treatment of asthma does not always comply with asthma guidelines (AG). This may be rooted in direct or indirect resistance on the doctors’ and/or patients’ side or be caused by the healthcare system. To assess whether patients’ concepts and attitudes are really an implementation barrier for AG, we analysed the patients’ perspective of a “good asthma therapy” and contrasted their wishes with current recommendations.

**Methods:**

Using a qualitative exploratory design, topic centred focus group (FG) discussions were performed until theoretical saturation was reached. Inclusion criteria were an asthma diagnosis and age above 18. FG sessions were recorded audio-visually and analysed via a mapping technique and content analysis performed according to Mayring (supported by MAXQDA®). Participants’ speech times and the proportion of time devoted to different themes were calculated using the Videograph System® and related to the content analysis.

**Results:**

Thirteen men and 24 women aged between 20 and 77 from rural and urban areas attended five FG. Some patients had been recently diagnosed with asthma, others years previously or in childhood. The following topics were addressed: (a) concern about or rejection of therapy components, particularly corticosteroids, which sometimes resulted in autonomous uncommunicated medication changes, (b) lack of time or money for optimal treatment, (c) insufficient involvement in therapy choices and (d) a desire for greater empowerment, (e) suboptimal communication between healthcare professionals and (f) difficulties with recommendations conflicting with daily life. Primarily, (g) participants wanted more time with doctors to discuss difficulties and (h) all aspects of living with an impairing condition.

**Conclusions:**

We identified some important patient driven barriers to implementing AG recommendations. In order to advance AG implementation and improve asthma treatment, the patients’ perspective needs to be considered before drafting new versions of AG. These issues should be addressed at the planning stage.

**Trial registration:**

DRKS00000562 (German Clinical Trials Registry).

**Electronic supplementary material:**

The online version of this article (doi:10.1186/s12890-016-0346-6) contains supplementary material, which is available to authorized users.

## Background

Asthma is a very common chronic condition that affects 5–7% of the adult population, with 235 million cases worldwide [1–3]. Despite the existence of regularly updated evidence based national and international asthma guidelines (AG), many asthma patients are sub-optimally treated and suffer from uncontrolled asthma [4–6]. To achieve the best care, the successful implementation of AG in general practice is essential, as the general practitioner (GP) is frequently the first to be contacted by concerned individuals, although most German patients have the possibility to consult a community based pneumologist directly. GPs manage, supervise and attend patients for long periods of their lives and guide them through the healthcare system. In Germany, the majority of asthma patients do not require constant specialist care and are regularly cared for by GPs, often within a structured, so called “disease management programme”(DMP), which is offered and strongly encouraged by all German statutory health insurance providers. Participation in the DMP is voluntary. The DMP is a three-monthly guideline based, structured follow up including lung function testing, review of symptoms and asthma related events, drug review and referral to a lung specialist or hospitalisation when predefined criteria are met. The DMP also advocates patients’ participation in asthma schooling programmes and provides information materials. German AG recommend special inpatient asthma rehabilitation programmes for severely affected patients at specialized centres.

Barriers to the successful implementation of all these guideline recommendations could potentially be based in the healthcare system, on the side of the doctors and/or patients. The published studies in this area almost exclusively address guideline implementation from the doctors’ viewpoints [7, 8]. The patients’ perspective is greatly underrepresented even though it is important to identify potential reasons for the patients to resist recommendations such as taking their daily corticosteroid as prescribed [9]. To identify such barriers, we performed an explorative study funded by the German Respiratory League (Deutsche Atemwegsliga) to investigate both patients’ and doctors’ concepts of good asthma treatment and to contrast them with one another and with guideline recommendations. In this paper, the patients’ views are presented. We discuss the emerging points in a larger international context [10, 11], focusing on patients’ expectations, concepts, experiences, beliefs, therapeutic goals and priorities as these may have impacts on AG implementation that are at least as substantial as those of physicians. The practicability and relevance of the AG (short form) and the GPs and GP- trainees knowledge of its contents were also assessed and have been published previously [12] (see Additional file 1: Figure S1 for project organisation).

## Methods

Qualitative methods, in contrast to quantitative approaches such as questionnaire based surveys, are used to investigate new fields of interest and generate hypotheses when nothing specific is known about a subject. Individual interviews and focus groups (FG) can both be used in this way. In this study we chose to employ FG, as a large amount of information can be collected quickly using this technique. All participants and their comments are given equal value, no matter how many participants agree with a given viewpoint. Moreover, via the snowball effect [13] patients inspire one another, encouraging each other to consider and verbalise a large range of prevalent opinions within the targeted population. The voiced ideas are immediately checked by the other participants and thus undergo a kind of direct evaluation.

FGs should be carried out until theoretical saturation is reached [14] and be limited to a maximum of 12 members to allow each person to participate in the discussion. Our FG had a time limit of two hours, including a 15 min break. Relevant themes were derived from a previous systematic literature research and compiled into a semi-structured discussion guide. When relevant themes did not arise spontaneously during the discussion, two cooperating experienced moderators used the open question technique to address them. The discussion guide featured the following topics:

- corticosteroids (inhaled and oral)
- the step up/step down principle of medication matching symptoms’ severity
- single substance- vs. fixed drug combinations
- non-pharmaceutical therapy
- components of asthma management
- illness related self-management
- sources used to gather information
- the asthma disease management programme (DMP).

To reflect reality as closely as possible, great care was taken to ensure diversity in the FGs according to FG recruitment recommendations. Younger and older, male and female patients were represented and in each FG at least one attendee participated in the DMP for asthma. With the aim of a maximal variance sampling, the FG participants were recruited from patients’ lists furnished by six GP practices; four in the city and two in the rural outskirts of Hannover. Inclusion criteria were a diagnosis of asthma in the patient’s records and age above 18. The patients were contacted in alphabetical order, starting with “A”. If the person of interest answered the phone and was willing to participate, his/her name was added to the final list of potential candidates. From this list, 10 to 12 consecutive patients (top down) were invited to the first fixed discussion date once written informed consent had been obtained, keeping a balanced distribution of gender and age. The remaining listed patients were contacted following the same procedure for the second and the next FG. When it became apparent that, due to cancellations at short notice, the first three FG contained too few men and younger participants, the recruitment was altered to a purposeful sampling. We also tried to distribute DMP participation and urban or rural residency as uniformly as possible between the groups. Written informed consent was obtained from participants of the focus groups.

The FG discussions were audio-visually recorded and transcribed. We used different qualitative and quantitative approaches to analyse this material in depth:

Based on the transcripts, thematic content analysis according to Mayring was performed by two independent scientists [15, 16], starting with the familiarisation with the data, coding separately, discussing and collating a code-book, reviewing the accuracy of the coding, defining themes and finally compiling a report.

Using MAXQDA®, sub-codes and coding lists were established, discussed and thereby more easily agreed on. The transcripts were re-evaluated using the agreed codes, differences were discussed until consensus was reached and themes summarised [17]. The results were compiled for each single FG and then pooled to be analysed and condensed on the next meta-level, thus identifying specific themes.

**Fig. 1:**
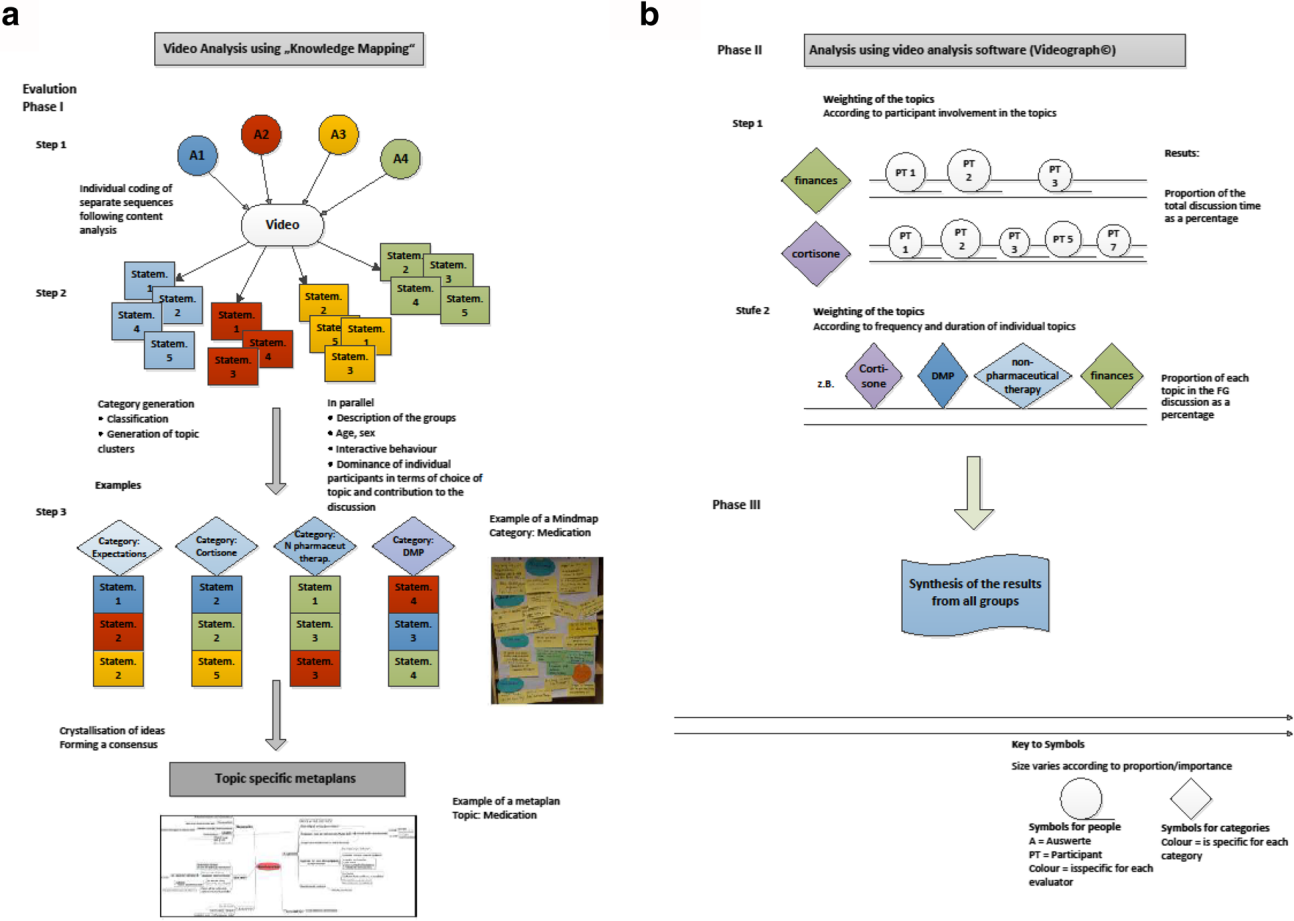
Graphical representation of the analysis steps. **a** Phase I, **b** Phase II and III

The video material from all the FG was directly analysed by a team of at least three independent scientists using content mapping techniques as shown in Fig. 1. The individually extracted content from consecutive 10 min sections of video tape was discussed between the participating scientists to identify ideas and issues, clustered and then summarised into themes on various meta levels (see Additional file 2: Figure S2) [18, 19]. This procedure allows rapid and detailed evaluation of the FG discussions. The first orienting analyses were therefore performed in parallel to continuing focus group data collection. This procedure served to facilitate responsive changes to the discussion guide when required.

The speaking time of each participant and the proportion of discussion time devoted to each specific topic were quantitatively analysed using Videograph®. This allows video material to be cut and recombined to calculate the duration of particular segments or sequences of interest.

## Results

The findings of the FG-discussions are presented in the following in three sections: the description of the participants of the five groups, the quantitative “time analyses”, and the themes addressed during the FG.

### Demographics

In total 37 asthma patients between the ages of 20 and 77 took part in the FG. Twenty-four of the participants were female (age range 20–67) and 13 male (age range 21–77). Interestingly, this final female/male ratio reflects that of the asthma prevalence in the adult population. All participants of FG IV were deliberately selected to be below the age of 30. In FG V only men participated (see Table 1). Every FG hosted at least one DMP participant and a mixture of urban and rural residents. Patients also came from a variety of professional and educational backgrounds, including students and retired people and individuals with and without a university education. They had received their diagnoses of asthma varying lengths of time before the study, some in childhood and others only a few weeks previously.

**Table 1:** Age- and gender distribution of FG I-V

		FG I	FG II	FG III	FG IV	FG V
Total		9	7	8	6	7
Women		7	7	7	3	0
Men		2	0	1	3	7
Age range (y)		20 – 71	27 – 51	44 – 77	21 – 27	47 – 77
Average age (y)		44.7	33.4	57.1	24	60.6
Time since diagnosis (y)	(m^a^)	16,2	17	13,4	9,9	25
	(sd^a^)	9,57	13,63	16,86	9,78	17,88
	(min^a^)	6	1	1	0,4	1
	(max^a^)	35	40	43	22	45

^a^mean (m); standard deviation (sd); minimum (min); maximum (max)

### Time analysis of the discussion themes

**Fig. 2:**
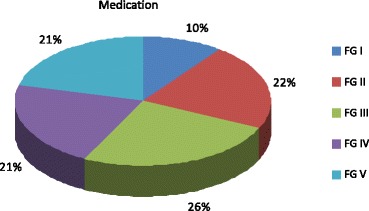
Proportion of the total time devoted to the theme “pharmaceutical therapy” in each FG (I-V)

The following themes were identified during the content analysis of the FG (see below): aims/expectations, pharmaceutical therapy, non-pharmaceutical therapy, DMP and Peak Flow, education/rehabilitation, coming to terms with the disease, the doctor-patient relationship, information sources, sports and the health care system. The amount of discussion time devoted to each individual theme varied considerably between groups and themes, as presented in Table 2. To visualise this variance, an example is shown in Fig. 2. It depicts the overall time devoted to the issue “medication” and the according percentage of time that each FG spent on this topic.

**Table 2:** Time spent on every topic in FG I-V in percent

Topic	FG I	FG II	FG III	FG IV	FG V
Aims & Expectations	38	22	7	13	12
Pharmaceutical therapy and cortisone	16	33	39	33	32
Non-pharmaceutical therapy	4	20	13	19	19
DMP and Peak Flow	7	6	6	4	4
Education & Rehabilitation	7	3	13	5	0
Handling the illness and doctor-patient relationship	3	7	6	7	9
Information sources	17	7	7	8	7
Sport	8	1	7	11	4
Healthcare system	0	0	2	0	13
**Total discussion time**	100% of 1 h 46’	100% of 1 h 34’	100% of 1 h 56’	100% of 1 h 55’	100% of 1 h 58’

The topics of medication and non-pharmaceutical therapy proved to be very time intensive in all groups 16–39% and 4–20% of the total time, while the topic of criticism of the system was only addressed in two of the FG and occupied less time overall (Table 2).

### Individual participants’ speaking times

The analysis of the speaking time of each individual showed that all participants took part in the discussions, albeit to different degrees and that none of the five FG was dominated by a single participant. Two examples are shown in Additional file 2: Figure S2.

### The topics addressed

The themes addressed in all five FG, both derived from the discussion guide (Table 2) and those that arose spontaneously, can be ranked from most to least time consuming as follows: pharmaceutical therapy and cortisone, aims/expectations, DMP, peak flow, non-pharmaceutical therapy, information sources, “handling the illness”, the doctor-patient relationship, sports and the healthcare system. (Translated examples of verbatim comments are presented in the Additional file 3: Table S1). This ranking does not imply any perception of descending order of significance by the participants.

The themes which were addressed during the five FG are detailed in the following in the order described above. Solely pharmaceutical and non-pharmaceutical treatments are placed consecutively for ease of comparison. At least one representative quote is added to every presented theme in order to illustrate the voiced opinions.

### Pharmacotherapy

Some patients described themselves as *“well managed”* (FG V) and were *“satisfied”* (FG II) with their medication. Many patients, however, attributed side effects to inhaled corticosteroids (ICS). Some reported negative experiences or talked about their fears (e.g. of getting a *“moon face”* FG II). Some participants expressed discontent and general doubts as to their doctor’s choice and dose of medication. Others perceived themselves as too dependent on their medication, particularly on *“emergency (reliever) sprays*”, and described this as restricting their daily life: *“When I notice that I don’t have my spray with me I immediately have an (asthma) attack out of fear” (FG IV).*

To prevent this “addiction” a great many FG participants do not take medication as prescribed*, “…because I don’t like medication” (FGII)* but alter the dose themselves according to their perceived disease burden. They do not inform their doctor before or afterwards and do not wait for feedback from their doctor before making changes to the dose *“I only take my medication when I feel bad” (FG III).*

Difficulties handling the inhalers were addressed in every FG *“… then also to perform the inhalation at exactly the right moment, without my tongue in the way, and and and…. So, it is already all complicated enough” (FG II).* In many cases, doctors apparently do not demonstrate or practice the inhaler use with their patients.

Most participants had never seen or possessed a written (emergency) medication plan. They also felt that explanations of the reason for the prescription of their medication and the impact and mechanism of action of the prescribed drugs were insufficient. Finding out about possible drug-interactions required individual initiative: *“I found out about the interaction of the anti-asthmatic drugs with my anti-hypertension medication all by myself”. (FG II)*

Almost all FG participants, regardless of age and sex, tried to use as little medication as possible. Views such as *“medication disrupts the [body] system” (FG IV)* were commonly voiced and accepted as a justification to stop taking any asthma drugs at all. The participants were in favour of a more personalised choice of single-agent drug and dosing, if medication was necessary, and liked the idea of simplifying its application as much as possible. However, only three participants had discussed the possibility of self-determined dose regulation (step up/step down principle) or a combined, individualised and symptom driven use of single-agent drugs with their doctors.

Younger patients described themselves as less rigorous in taking and (correctly) using the medication than older patients. Taking medicine was perceived as onerous and moreover “*the spray has a disgusting consistency” (FG IV).* Some of the older participants however, particularly older women, tried hard to follow the prescription in every possible detail.

### The topic of corticosteroids

Many of the FG participants had a negative image of corticosteroids (“cortisone”). They expressed worries or fears about harm from long term use and/or were afraid of drug dependency. For this reason some participants stopped or restricted all inhalation of drugs that they thought contained cortisone, according to the expressed belief that *“less is still enough*” (FG I). The prescribed drugs were however used on a regular basis if the word corticosteroid/cortisone was not prominent in the package information or not recognised as an ingredient due to the use of the exact name of the chemical active component e.g. Fluticason-17-propionat. The patients also stated their awareness that no other treatments are as immediately effective as corticosteroids and that despite their concerns they do nevertheless use this medication. *“You know that cortisone is not good for the body – but it helps.” (FGI*)

### Non-pharmaceutical therapy

Many, mostly female and younger, participants emphasized that they proactively searched for additional *“helpful*” (non-pharmaceutical) treatments. They stated that they were trying to *“develop a feeling for your own body” (FG II)* and to use this knowledge to treat themselves. Their search was mainly driven by the feeling that their doctor did not acknowledge and treat them *“in their totality*” (*“Please consider me holistically” (FGII)*). The participants were happy to talk about the options they had identified and experienced as helpful: Physical activity and sports were perceived as useful in the attempt to reduce medication and were therefore performed by many participants. Two main points were mentioned as troubling with the *“sports”* approach: what to do when the available and offered special lung sports activities did not suit or complement personal sport preferences, was an issue addressed mainly by younger and male participants. Fear of being unable to perform sufficiently well in a sports group for non-asthmatics prevents joining groups for team sports, as a female participant explained.

With few exceptions, participants said that their doctor had not discussed specific exercise possibilities or physical activity in general. Apparently sports were more likely to be recommended by other asthma patients. Finding and practising a sport was mostly a result of the patient’s own initiative rather than of advice from their GP. Relaxation and breathing exercises were experienced as helpful by many of the participants who felt that the surge of an asthma exacerbation could be *“prevented” (FG I)* this way. The necessary techniques and practice were mostly acquired through the patients’ own initiative or during a (pneumological) rehabilitation program, rarely via the DMP.

Complementary or alternative therapy approaches were strongly advocated by some of the participants, although labelled as *“too time consuming*”. Acupuncture, traditional Chinese medical teas, applications of cold water and foot reflexology were mentioned and partly discussed extensively.

In all five FG the wish to integrate non-pharmaceutical treatment in their routine treatment was expressed and strongly supported. Some participants emphasized that they wanted to be “*considered holistically*”, expressing the desire that their doctor would explicitly consider all their complaints and their mutual implications and look for an appropriate treatment for all of them.

Avoidance of allergens and advice on smoking cessation or avoidance, weight reduction support, psychotherapy and help in scheduling medical appointments were wished for during the FG discussions. Some participants also voiced their desire that doctors might help to organise the financial aspects of the therapy, particularly for purchases needed for a stay in a rehabilitation clinic, for prescription fees and for costs of complementary and alternative treatments not covered by health insurance.

### Dealing with the illness and medical advice on daily life

All participants of the FG had already experienced exacerbations and were afraid of the next one. Although some were aware of the triggers, implementation of allergen avoidance seemed to be a problematic issue*: “I’m not giving my cats away, they are part of my life – I would rather have asthma” (FGIV).* The recommendation of giving away pets met with strong resistance from younger participants. Physicians’ recommendation that *“asthmatics should not do the cleaning” (FG I)* and dusting in the household was criticized as being very unrealistic. Despite their fear of sudden “asthma attacks” and the feeling of unpredictability, not all participants had their “emergency sprays” readily accessible or could show them on demand.

Ways of dealing with illness ranged from *“getting used to it over time” (FG IV)* and resigned acceptance to the attitude that *“asthma is not a life-determining factor” (FG I).* The last mentioned point of view was commonly expressed by patients under 30, as were denial, rejection and anger. Younger FG participants were more likely to try to ignore their illness, while older patients were often very interested in its etiology and therapy options.

The extent of each individual’s own responsibilities was largely discussed in FG I and II. Some patients pointed out that doctors should encourage patients to take responsibility for their own health and use this as a “treatment resource”. Others felt that too much responsibility was placed on the patients already, and that the healthcare protagonists were thus *“shirking the duty of care”* (FG I).

### Experiences with DMP

Some FG participants were not participating in the asthma disease management programs (DMP). Some even stated that they had never heard about DMP before the FG discussion. Positive effects of DMP mentioned by the DMP-participants were that *“examinations were now made twice, by two doctors*” (FG II) (meaning GPs and specialists), regular doctor’s appointments were known in advance and information material was sent free of charge (and without request) to their homes. However, several participants added that they never read the information material sent by the health insurers.

The Peak Flow meter device was unknown or its use was judged as nonsense by some FG-participants. Sometimes measurements were avoided because the results *“are frightening” (FG V)*. Mostly male participants were very fond of the opportunity to measure and draw figures with the findings, however without deducing personal consequences for their use of drugs. The reason for this behaviour, while certainly interesting, was beyond the scope of these FGs. In some cases the figures were used for “*own estimation”* (FG V) of their treatment or for “*reassurance*” (FG II) to confirm that the medication is of high quality.

### Views on the topic of rehabilitation

The few participants who had participated in a hospital based inpatient rehabilitation programme before the FG discussions referred to it in positive terms and considered it helpful. However some critical comments were added: lack of time as a barrier to in-patient rehabilitation was described by both self-employed and employed participants. Some complained about the expenses entailed by in-patient rehabilitation, as they felt prompted to buy a suitcase, new pyjamas etc. while lacking the necessary financial resources. The geographical location of the rehabilitation centres was discussed in detail, patients feared that some were likely to “worsen asthma” (FG II) due to the local climate.

Participants who were at the start of their professional careers or employed on temporary contracts complained about the required length of rehabilitation. They feared that the three weeks required by the health insurance companies would endanger their employment or professional advancement. They also criticised the activities offered during the rehabilitation programmes, which they thought were conceived for elderly people, and pointed out the lack of peer groups in the rehabilitation facilities. Rehabilitation programmes are *“…only for children and old people.” (FG IV)*

The concept of outpatient rehabilitation, which is very infrequent in Germany, was largely unknown. Information about it was received with keen interest, particularly by the participants critical of inpatient rehabilitation programmes.

### Experiences with information sources about asthma

Participants who had taken part in an asthma schooling programme were very happy with what they had learnt. They regretted, however, quickly forgetting the lessons learned without the opportunity to “*refresh the information*” (FG I). Further information sources mentioned were printed media, especially a (non-professional, non-scientific) “pharmacy journal” (Apotheken Umschau) targeted at the general public and distributed in pharmacies free of charge. Discussions with acquaintances and relatives were perceived as even more important.

Searching the internet was judged very critically: participants talked about an overwhelming “*flood of information*” (FG II) and complained about the difficulties in filtering and evaluating the results, stating that without informed assessment *“you just get worse”* (FG II) by reading all contributions.

Brief information in printed form was largely preferred to electronic data, even by the younger participants, although books were often perceived as too complicated, too long and time consuming, or as using an incomprehensible language: *“Then I could throw this book into the corner, really hard” (FG II)*.

Oral information from their doctor was rated highest by the FG-participants. It seems to be well accepted and considered to be the most reliable.

### Doctor - patient relationship

Many patients reported a lack of consensus between different doctors in terms of diagnosis and best treatment which made them feel concerned. Disagreements e.g. on appropriate medication, or lack of communication between GPs and specialists, e.g. lacking reports on the outcomes of consultations, were pointed out as problematic and addressed in all the FGs. The participants made clear that they did not want to take sides and expressed their fear of offending one doctor, e.g. their GP, by consulting another doctor, e.g. a pneumologist, for a second opinion.

No one recalled real shared decision making processes. Many participants voiced the feeling of not being acknowledged as an expert on their own illness experience and their life with asthma. They wished to be involved in the choice between several possible therapies, and to share the decision-making process with their doctors: they appealed to *“…treat the patient as a well-informed and responsible person.” (FG II)*

### How the healthcare system is perceived

Treatment differences between privately and statutorily insured patients were criticised, especially with regard to waiting times and choice of medication*.* Despite the almost complete financial coverage available in Germany, some FG attendees complained about insufficient financial support provided by the (public/statutory) healthcare system and feared inadequacy of care. *“Good care depends on luck*” was an idea common to all FG as *“not every GP has pulmonary function test equipment and* (even when he possesses the equipment) *can make accurate diagnoses” (FG V).*

Some FG participants felt as if doctors interpreted their complaints as *“attempts to fool the health care system or to take undue advantage of it” (FG V)*, and expressed feeling helpless, ignored and misunderstood by the doctors concerned. To these participants, it seemed that doctors rather work in the interests of the public authorities than in the interests of the patients (which made the FG participants angry).

### The topic of time requirements

Time seemed to be an important issue for patients dealing with asthma in several different ways: (1) FG participants claimed that they did not have enough time for the recommended therapy: participation in a schooling programme or performing relaxation exercises was perceived as demanding considerable time. (2) Participants complained that there was no way to get a doctor’s appointment quickly and when needed, and (3) about having to spend a lot of time in waiting rooms. *“A doctor’s appointment takes time” (FG IV)* (4) Time absent from work had to be excused and tolerated by colleagues and employers alike. (5) The brevity of the appointment with the doctor was highlighted in all the FG, as there was hardly any or no time at all to discuss difficulties, symptoms and problems or to ask for individual advice.

Doctor’s appointments were avoided by some of the employed younger participants. The quarterly check-up required by the DMP was criticized for requiring too frequent absences during working hours, which, in addition to potential emergency appointments in case of exacerbations, put them at risk of losing their jobs.

### Private money and public budget

Access to financial resources was perceived as a limiting factor for good asthma therapy. Some of the FG participants had insufficient means to afford alternative or complementary treatments not covered by health insurance which they nevertheless believed to be helpful. Out-of-pocket drug fees as imposed by German public health insurance at the time of the FG performance were perceived as a financial strain for chronically ill patients in general, and care covered by insurance was declared to be insufficient.

Additionally, less costly generic drug preparations which were prescribed or dispensed (according to the rules of statutory public health insurance) were judged to be ineffective: *“Aut idem* [generics] *is as good as a mouthful of water” (FG IV).* From the point of view of the statutorily insured patients, privately insured patients received appointments more quickly and received more effective medication. They believed that corresponding to the higher consultation fees, more expensive, sophisticated and more effective drugs would be reimbursed by the private insurance companies, while *“as a publicly insured patient I only got crappy penicillin” (FG II).*

### Differences between the focus groups due to age and sex

The younger patients (FG IV) were ready to deny having a chronic disease, the therapy or the impact of both on their daily life. If possible they would prefer to forget completely about asthma and certainly didn’t liked to be reminded by friends or relatives of their “handicap”, detesting looking for information or being informed about the illness, its possible progression, treatment-options or consequences. Young asthmatics didn’t show any interest in improving their therapy, when it was perceived as sufficiently successful, while elder asthmatics didn’t seem to have these acceptance problems. These findings correspond to and explain the adherence problems Koster et al.[20] have identified in the group of young patients with asthma. Serious concern about supplemental days off work due to asthma was very present in FGIV and voiced only by young asthmatics.

Female participants were more interested in and positive about complementary medicine, and more active in seeking information from multiple sources. In contrast, male participants mostly named their GP as their only source for information. The fondness for drawing graphs was also a male characteristic, though often there seemed to be no consequences in terms of their treatment. Male patients reported being confronted by physicians who were suspicious of a purposeful mismanagement of their illness with the aim of an early retirement from professional life.

## Discussion

As described by Pelaez et al. [21], patients’ adherence to asthma guideline recommended treatment must be considered on several levels; the patients themselves, the doctor-patient relationship and the health care system. We have previously investigated doctors’ knowledge of the AG [12] and here we focus on the patients’ motivation and wishes in the search for relevant barriers to a more successful AG implementation and therefore to effective evidence based treatment of asthma patients. Our results generated insights into the patients’ perspectives and their wishes with respect to a “good treatment” for their chronic condition. Their concerns with regard to the requirements of the day-to-day management of asthma “in an unpredictable world”[22] and the areas to which there is resistance, as presented here, provide a foundation for the development of strategies to overcome opposition to guideline based treatment.

Some topics were particularly prominent:

**Reducing medication** to an absolute minimum was the most frequently mentioned goal, particularly with regard to “cortisone” which had negative connotations for participants. Despite this, most of the FG participants shared the conviction that corticosteroids are “unfortunately” the “only” medication that can really help. Although this appraisal was undisputed in all FG, sometimes both viewpoints of rejection and dependence were expressed by the same person, showing the smouldering conflict between emotions and intellectual understanding.

Fear of cortisone seemed to be partly based on lack of knowledge of the selective local action of inhaled corticosteroids. This disinformation and anxiety may lead to the patient reported changes made to medication without informing their doctor. Concerns about the safety of asthma drugs have also been reported internationally as barriers to AG implementation [11, 23, 24]. Although other studies suggested only a minimal influence of the fear of cortisone on patient compliance [25, 26]], our FG participants expressed a fear of drug dependency as well as fear of a long term loss of effectiveness of the only drug that helps in very frightening emergencies. Both reasons contribute to a restricted and non-compliant use of corticosteroids. Bender et al., however, discussed patients’ convictions that their asthma was not bad enough to require daily inhalative therapy; a similar belief to that noted by Horne et al., that, in the absence of symptoms, the need for treatment is no longer perceived [11, 24]. The sceptical attitudes of our patients towards corticosteroids resemble the pattern of behavior described in a Danish study by Al-kalemij et al. in which the patients stated that they did not regularly take their ICS as they noticed no effect [27].

**Rehabilitation** was considered important but difficult to implement for employed/working patients who were concerned about the threat of losing their jobs. Nevertheless, comprehensive advice and support and also the extension of training opportunities and out-patient rehabilitation possibilities would possibly be helpful. Schweikert et al. showed that in- and out-patient rehabilitation treatments were of equal benefit for patients following acute coronary illness [28]. Bingisser et al. assessed an inexpensive model of out-patient rehabilitation programme for patients with asthma and COPD in Switzerland and demonstrated a positive effect on physical capacity and quality of life [29].

The FG participants in our study called for **improved communication,** not only with the doctor but also between GPs and specialists, which they considered important for their safety and reassurance. Usually German patients are supposed to see their GP first in order to be referred to a lung specialist if needed. Lung specialists should send the patients back to the GP adding written advice on the treatment to be followed. When deviations occurred from this procedure, the FG patients felt caught between two opposing forces and impelled to act as an intermediary between their doctors, which was described as a very uncomfortable position. These findings match those of an international study from Newcomb et al. [30], which also showed that asthma patients were dissatisfied with communication with and between healthcare workers. However, as also seen in our study, these patients rarely addressed their questions directly to healthcare workers. Patients in our FG expected to be provided with extensive information without having to ask, although they were aware that healthcare providers had little time to talk to them.

Participating patients perceived more time with the doctor to be sine qua non conditions for good asthma therapy, and wanted extensive information about all aspects of therapy and lifestyle.

Differences in perception of the value of pharmaceutical and non-pharmaceutical therapies by patients and doctors seem to be obstacles to better guideline implementation in daily practice [10]. The FG participants had the impression that their doctor informed them exclusively about medication, without discussing all of the other therapeutic options mentioned in the 13 pages of information issued by the German Respiratory League AG [31]. These include e.g., psychotherapeutic support, help with lifestyle adjustments such as regular exercise, and in some instances complementary medicine approaches. Finding and practising an appropriate sport was mostly a result of the patient’s own initiative rather than of advice from their GP, although the AG strongly recommends that doctors encourage their patients to exercise in spite of their asthma. This does not mean that patients would follow medical advice at all costs, when received. As Clark [32] pointed out, all management efforts can be enhanced or impeded by the social and physical environment of the patient. To some FG-participants keeping their pets was more important than avoiding asthma exacerbations. One could wonder whether intensified and appropriate communication would be able to overcome similar harmful attitudes.

Advice was wanted in the areas of complementary medicine approaches, psychological support and how to enhance their own daily capacity to help themselves in choosing, adhering to and applying therapeutic measures. Although some patients described themselves as “well managed” and were satisfied with their medication this however doesn’t generally mean that they were well controlled according to GINA Guidelines, since patients trend to overestimate their asthma control [33–35].

The lack of **written medication plans** reported in our FG has also been found in other studies [23, 36]. Sheares et al. strongly advocates personalizing interventions in this context [37]. Despite differences in focus and in the techniques used, Haughney et al. also found that a personal asthma action plan was wanted by the patients [23]. Such plans were recommended in the asthma guidelines and will be noted and implemented in the electronic patient documentation systems in Germany in 2016.

The step up/step down principle of medication was completely unknown to the FG participants and clearly needs some more publicity.

One of the most important factors attributed to problems in finding and applying appropriate treatment was a **lack of time,** for both doctors and patients. Apart from cortisone, time seems to be THE topic when dealing with an asthmatic condition. Doctors’ appointments were not available quickly enough and medication was therefore changed without consulting or telling the doctor – despite the fact that waiting times for appointments in Germany are short compared to other countries [38]. Alleviating breathing techniques were not performed by the patients and offers of educational courses were also not taken up due to lack of time. Doctors apparently do not take time to demonstrate or practise the inhaler use with their patients. They also seem not to give the resulting adherence problems any further thought for the same reason: lack of time. Our patients’ views confirm the findings of Schubert et al.[39] showing that although doctors are aware of guideline implementation problems, they also blame “time pressure”, which prevents them from discussing guideline recommendations and non-pharmaceutical therapy options with their patients.

**A lack of financial resources** was seen by some participants as a factor limiting good asthma treatment. These worries were related to the ability to access alternative therapies and also to dissatisfaction with being restricted to treatment with generic drug preparations. According to current German legislation, generic drugs should be prescribed due to their lower costs in comparison to brand name originals. Concerns relating to the costs of the disease and an “unequal distribution” of resources were also described by Bender et al.[11], while Magzamen et al. assumed there is an implicit competition between good health and other needs for both time and income, a topic our participants were also aware of [22].

Participants of the FG expressed the desire to act on their own initiative or take responsibility for their therapy, but were unable to define precisely to which degree this should happen. Barner et al. assessed (young) asthma patients’ willingness to pay for and invest time in an asthma self-management program. It seems that patients exhibiting suboptimal behaviors during asthma attacks, although having greater (perceived) access to health care resources were willing to pay more than the $29.50 average and spend 5.8 h a week in the educational program [40]. With respect to **disease related self-management**, our results suggest that some of the patients fitted the therapy to their needs and expectations, with or without consulting a doctor, which could potentially have direct and indirect negative outcomes. An immediate worsening of symptoms could result, or, indirectly, a reduction in the doctor’s ability to interpret the effects of treatment given may occur. Similar behavior was observed by Al-kalemji et al.[27]. The desire for more support (from a doctor) in order to develop competence in disease related self-management was seen in all our FG. Gibson et al.[41] however showed that a simple transfer of knowledge is not sufficient to achieve good management; practice in the necessary skills, for example, is also required [11, 39].

The methods applied in this study have some limitations; social desirability bias is a potential problem in qualitative research, both in FG and individual interviews, which must be reduced by the moderators in order to maximize the number, diversity and reliability of expressed opinions. In this respect the moderators must ensure that an atmosphere of trust is created so that even timid individuals feel able to voice their opinion without fear of negative comments. Of course all participants have to be assured of anonymity. The participants must also have no social connections to one another.

The results of our qualitative FG study offer an insight into aspects of guideline implementation that are relevant to patients; however they cannot be considered as representative of the whole German population of asthma patients. During the recruitment of participants to the FG, efforts were made to maximize variance of the sample. As participation was voluntary, some patient groups with specific concerns were probably under- or not at all represented. Children and adolescents and their parents, for example, who were not our target group, are likely to have specific needs. Patients unable to independently access the study center or patients with limited language ability were not invited to participate and hence are also not represented. Moreover, due to the recruitment of participants from GP practices, it cannot be excluded that, as described by Bender et al.[11], that patients from low socioeconomic groups were underrepresented as they tend to underuse the health care system. We suggest that this group should be explicitly considered in further studies, as it faces particular challenges.

Nevertheless, results from international studies support the assumption that our findings comprise the essential aspects of the patients’ perspectives on asthma treatment and barriers to guideline recommendation. The participants in our study do not entirely reject an evidence based therapy in line with guidelines, as the findings of Cabana et al.[10] relating (physicians perceived) patient barriers suggest. However they emphasise and accentuate different priorities, calling for a stronger focus on a holistic treatment approach and also non-pharmaceutical therapy recommendations, which should be indicated and discussed during doctor’s appointments.

In this paper we discuss patients’ beliefs and the barriers they have experienced to optimal asthma treatment. Some points of view, such as fear of cortisone, complaints about out-of-pocket costs or the desire for reimbursement for complementary medicine treatments could be addressed via improvements to current patient education programs, thus correcting misunderstandings leading to commonly held false beliefs. The identification of these “teachable areas”, which are currently not included in patient education programs, is an added value of this paper.

Another important aspect of our findings is the identification of clear and realistic improvements that could be made to current standard primary care in Germany, e.g. the introduction of written plans (on its way), shared decision making, inhaler choice and training [42]. Moreover several potential interventions have been defined, which should be addressed at the level of the GPs.

These findings are also likely to be applicable to other diseases. Patients with asthma are frequently multi-morbid, meaning that their reactions to other disease guidelines are likely to be similar to those shown here. To develop the findings shown here further, individual interviews and questionnaires to determine the representativity of the various voiced opinions could be added in the future.

(1) two different classes of intervention (for patients and for doctors) should be developed to improve guideline implementation and (2) guidelines need to be evaluated in the context of the setting of delivery [10, 37].

## Conclusion

This study documents barriers to the implementation of asthma guidelines during daily life, from the patients’ perspective. Some of the ideas presented here can and should be implemented, such as improvements to doctor-patient and doctor-doctor communication, holistic therapy and individual and personalised counselling. Doctors should use the findings of our survey and engage in a more explicit shared decision making process with their patients regarding therapy options.

Other suggestions of the focus groups are currently not practicable, but nevertheless represent important goals for future developments, such as alternatives to cortisone with reduced side-effects. We consider it sensible and essential to document possible resistance, expectations and wishes from the side of affected patients before releasing new treatment guidelines. Concerned individuals should already be involved in the guideline developing process at an early (preparation) stage; a requirement that is now also met on a national level by the “Arbeitsgemeinschaft der Wissenschaftlichen Medizinischen Fachgesellschaften (AWMF)” and internationally by patients’ participation initiatives [35, 43].

Future development of guidelines will hopefully include patient representatives and knowledge of patient perspectives in order to make recommendations that fit better with the (multiple) realities of patients’ daily lives.

## Additional files

Additional file 1: Figure S1. Organisation flowchart of the whole project. (DOCX 44 kb)
Additional file 2: Figure S2. Individuals’ speech times In FG I and II. (DOCX 39 kb)
Additional file 3: Table S1. Translated examples of verbatim comments for the indicated focus group topics. (DOC 1.10 mb)

## Abbreviations

AG: Asthma guidelines
AWMF: Arbeitsgemeinschaft der Wissenschaftlichen Medizinischen Fachgesellschaften
COPD: Chronic obstructive pulmonary disease
DMP: Disease management programme
FG: Focus group
GP: General practitioner
ICS: Inhaled corticosteroids
