# Supplementary figures and images for: What patients really think about asthma guidelines: barriers to guideline implementation from the patients’ perspective
Source: BMC Pulm Med. 2017 Jan 11;17:13. doi: 10.1186/s12890-016-0346-6 (PMC5225589; doi:10.1186/s12890-016-0346-6)

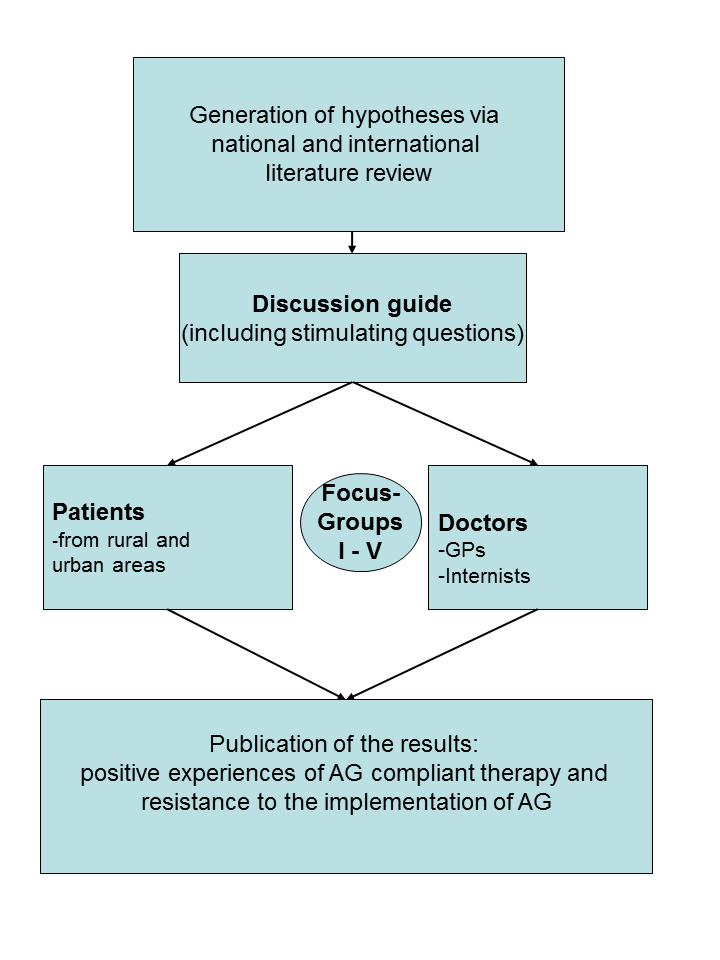

Supplement: Additional file 1: Figure S1. — Organisation flowchart of the whole project. (DOCX 44 kb) [file 12890_2016_346_MOESM1_ESM.docx]

Supp. Fig.3: Translated examples of verbatim comments for the indicated focus group topics


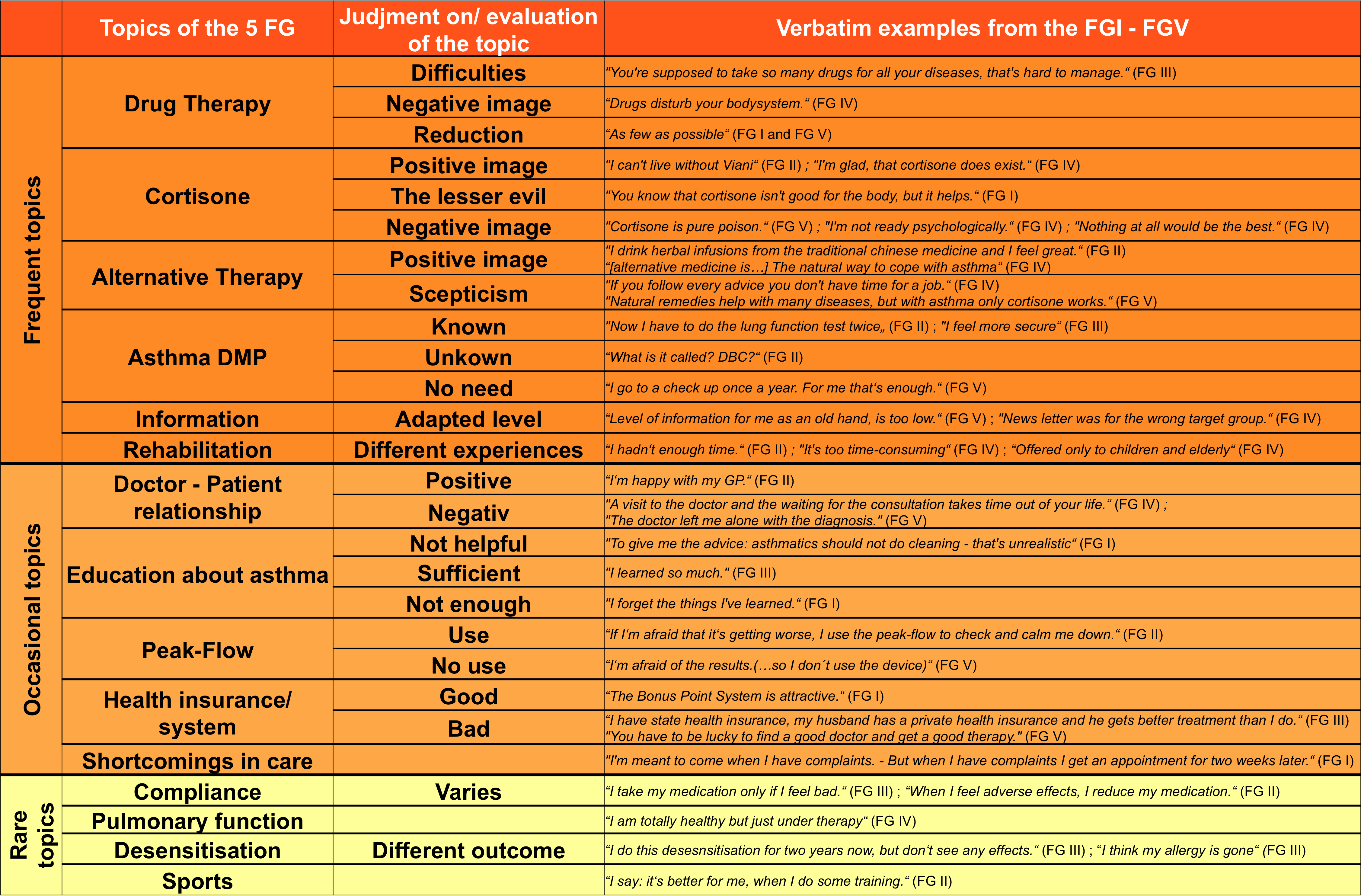

Supplement: Additional file 3: Table S1. — Translated examples of verbatim comments for the indicated focus group topics. (DOC 1.10 mb) [file 12890_2016_346_MOESM3_ESM.doc]
