# Supplementary material for: What patients really think about asthma guidelines: barriers to guideline implementation from the patients’ perspective
Source: BMC Pulm Med. 2017 Jan 11;17:13. doi: 10.1186/s12890-016-0346-6 (PMC5225589; doi:10.1186/s12890-016-0346-6)
Supplement: Supplementary file 2 [file 12890_2016_346_MOESM2_ESM.docx]

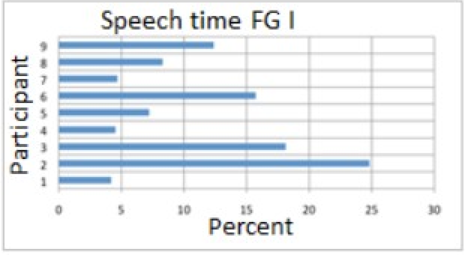
Speech time FG I
Participant
Percent
1
2
3
4
5
6
7
8
9
0
5
10
15
20
25
30

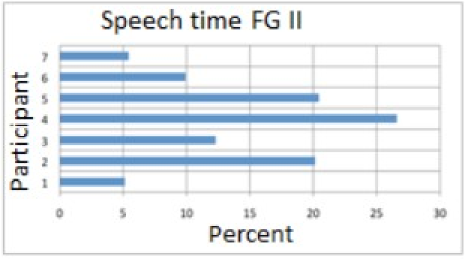
Speech time FG II
Participant
Percent
1
2
3
4
5
6
7
0
5
10
15
20
25
30
